# What can we learn from China’s health insurance reform to improve the horizontal equity of healthcare financing?

**DOI:** 10.1186/s12939-022-01793-3

**Published:** 2022-12-01

**Authors:** Fan Yang, Mingsheng Chen, Lei Si

**Affiliations:** 1 School of Health Policy & Management, Nanjing Medical University, No. 101, Longmian Avenue, Nanjing, 211166 China; 2 Center for Global Health, Nanjing Medical University, Nanjing, China; 3 School of Health Sciences, Western Sydney University, Campbelltown, Australia; 4 The George Institute for Global Health, University of New South Wales, Kensington, Australia

**Keywords:** Horizontal inequity, Redistributive effect, Healthcare payment, Health insurance, Financing

## Abstract

**Background:**

Universal health coverage is a challenge to horizontal equity in healthcare financing. Since 1998, China has extended its healthcare insurance schemes, and individuals with equal incomes but different attributes such as social status, profession, geographic access to health care, and health conditions, are covered by the same health insurance scheme. This study aims to examine horizontal inequity in the Chinese healthcare financing system in 2002 and 2007 using data from two national household health surveys.

**Methods:**

Multi-stage stratified random sampling was used to select 3,946 households with 13,619 individuals in 2002, and 3,958 households with 12,973 individuals in 2007. A decomposition method was used to measure the horizontal inequity and reranking in healthcare finance.

**Results:**

Over the period 2002–2007, the absolute value of horizontal inequity in total healthcare payments decreased from 997.83 percentage points to 199.87 percentage points in urban areas, and increased from 22.28 percentage points to 48.80 percentage points in rural areas. The horizontal inequity in social health insurance remained almost the same in urban areas, at around 27 percentage points, but decreased from 110.90 percentage points to 7.80 percentage points in rural areas. Horizontal inequity in out-of-pocket payments decreased from 178.43 percentage points to 80.96 percentage points in urban areas, and increased from 26.06 percentage points to 41.40 percentage points in rural areas.

**Conclusion:**

The horizontal inequity of healthcare finance in China over the period 2002–2007 was reduced by general taxation and social insurance, but strongly affected by out-of-pocket payments. Increasing the benefits from social health insurance would help to reduce horizontal inequity.

## Background

Since 1978, the planned economic model has been gradually replaced by a market-oriented model in China. The influence of the market model has now reached every corner of Chinese society. In urban areas, traditional health insurance plans, the Free Medical Service and the Labor Medical Service, were collapsed due to financial pressure on paying for the medical bill. In rural areas, the Cooperative Medical Scheme (CMS), which used to provide to health cover for the residents of rural areas, was rapidly collapsed due to rural economic reform and the implementation of the household contract responsibility system at the beginning of the 1980s [1].

China began to redevelop its health insurance schemes at the end of 1998 (Table 1). The Urban Workers Basic Medical Insurance (UWBMI) covers public sector workers and retirees in urban areas (for example, from government departments, and state-owned and collectively-owned enterprises). Both employers and employees paid contributions to UWBMI, of about 6%–8% and 0%–2% of the employee’s salary. UWBMI was gradually extended to cover non-public sector workers, including migrant workers and workers in private enterprises, private non-enterprise units, social organizations, and foreign-invested enterprises. UWBMI coverage increased from 30.4% of urban residents in 2002 to 44.2% in 2007 [1, 2]. In 2003, the New Cooperative Medical Scheme (NCMS), an initiative to rebuild health insurance and overhaul the healthcare system in rural areas, following the gradual dissolution of CMS from the late 1980s. Since its formation, China’s authorities have provided additional public spending for NCMS, which has achieved a high rural coverage level with the percentage of rural residents insured increasing from 9.5% in 2002 to 89.7% in 2007 [1, 2] (see Table 1).

**Table 1:** Changes in China’s social health insurance schemes between 2002 and 2007

	**2002**		**2007**	
	**UWBMI**	**CMS**	**UWBMI**	**NCMS**
Target population	Formal urban workers in public sectors, such as government department, the state-owned enterprises and collectively-owned enterprises	Rural residents	Workers in public sectors; workers in private enterprises, social organizations, foreign-invested enterprises; migrant workers, etc	Rural residents
Coverage	30.4% of the total urban residents ^a^	9.5% of the total rural residents ^a^	44.2% of the total urban residents ^a^	89.7% of the total rural residents ^a^
Risk-pooling unit	City	Village or town	City	County
Financing source				
Government subsidy per person	Nil	Nil	Nil	20–50 RMB Yuan ^b^
Employer contribution	6%–8% of salary	\	6%–8% of salary	\
Individual contribution	0%–2% of salary	NA^c^	0%–2% of salary	10–20 RMB Yuan ^b^

*UWBMI* Urban Workers Basic Medical Insurance, *CMS* Cooperative Medical Scheme, *NCMS* New Cooperative Medical Scheme, *NA* Not available

^a^Data source: Reports from the third (2002) and fourth (2007) National Health Services Surveys

^b^Data source: China Statistical Handbook of NCMS 2007

^c^No official record of individual contributions, but they were flat-rate

The healthcare financing system has therefore been extensively reformed and the population coverage of health insurance schemes has considerably improved. There is, however, a potential threat to the horizontal equity of healthcare finance. Financing equity of healthcare depends on both vertical and horizontal equity [3, 4]. Vertical equity implies that people having greater economic ability ought to pay more and horizontal equity implies that people with equal economic ability should pay the same. The issue of vertical equity and its progressivity have been studied by many researchers [5–8], but very few have examined horizontal equity of healthcare financing [4, 9–11]. Horizontal inequity is increasingly recognized as an important component in the healthcare financing system, and plays a key role in adjusting the economic rank order of the general population when a relatively high healthcare payment occurs. China’s health insurance schemes have expanded health coverage to individuals with different socioeconomic status. In each socioeconomic group, individuals may also live in either urban or rural areas, and have different health conditions, social status, and access to healthcare. Much uncertainty still exists about the relation between horizontal inequity and the expansion of health insurance schemes. This study therefore aimed to examine the horizontal equity of healthcare finance in 2002 and 2007 in one province of China, to shine new light on the question of how well different forms of healthcare financing performed after reform of China’s health insurance system.

## Materials and methods

### Data source

The unit of analysis used was households. Two rounds of household survey were conducted in China’s Gansu province in 2003 and 2008. These surveys recorded basic household information and healthcare use of household members in 2002 and 2007. Gansu is located in the northwest of China and is an impoverished province with a population of more than 26 million [12]. The survey randomly collected data from 13 counties or county-level cities using multi-stage stratified random sampling. Eight communities or administrative villages were sampled in every city or county. About 30 households from each community or administrative villages were then randomly sampled, giving a total of 3,946 households (1,974 urban and 1,972 rural) containing 13,619 individuals (5,880 urban and 7,739 rural) in 2003 and 3,958 households (1,979 urban and 1,979 rural) containing 12,973 individuals (5,581 urban and 7,392 rural) in 2008 (see Tables 2 and 3).

**Table 2:** Descriptive statistics and socioeconomic characteristics for the urban sample

Variable	2002			2007		
	Obs	Mean	Std. dev	Obs	Mean	Std. dev
Gender						
Male	3283	0.56		2755	0.49	
Female	2597	0.44		2826	0.51	
Age, years	5880	37.47	20.18	5581	40.44	20.83
0–14	984	7.47	4.43	788	7.72	4.28
15–44	2705	30.96	8.10	2469	31.85	8.75
45–59	1157	51.73	4.30	1142	51.77	4.25
60 +	1034	67.08	5.73	1182	69.23	6.14
Average number of household members	1974	3.46	1.47	1979	2.93	1.14
Equivalent household expenditure	1974	5924.62	6706.05	1979	8855.68	5830.61
Equivalent household OOP expenditure	1974	719.84	1852.17	1979	1184.40	2581.13
Health insurance						
None	3886	0.66		1918	34.37	
Any	1994	0.34		3663	65.63	

Data source: Authors’ calculations from 2003–2008 NHSS data

**Table 3:** Descriptive statistics and socioeconomic characteristics for the rural sample

Variable	2002			2007		
	Obs	Mean	Std. dev	Obs	Mean	Std. dev
Gender						
Male	4674	0.60		3657	0.50	
Female	3065	0.40		3735	0.50	
Age, years	7739	30.75	19.53	7392	33.65	20.34
0–14	2107	7.75	4.32	1711	7.92	4.49
15–44	3608	29.54	8.22	3455	30.49	9.32
45–59	1345	51.39	4.13	1336	51.88	4.33
60 +	679	67.70	6.35	890	68.02	6.26
Average number of household members	1972	4.97	1.95	1979	4.22	1.42
Equivalent household expenditure	1972	2483.21	8740.46	1979	3550.68	2767.75
Equivalent household OOP expenditure	1972	388.56	1516.20	1979	506.68	963.98
Health insurance						
None	6677	0.86		398	0.05	
Any	1062	0.14		6994	0.95	

Data source: Authors’ calculations from 2003–2008 NHSS data

The survey was administered via household interviews. All household members aged 15 years or more were interviewed by trained data collectors in each sampled household. Incapacitated people and children under 15 years old were interviewed through their guardians. The face-to-face interviews, implemented by trained data collectors, were done with a structured questionnaire, containing a series of questions regarding household’s demographic and socioeconomic characteristics, including household expenditure, number of family members, urban–rural classification, and gender, age, educational attainment, and employment type of household members. Monthly household expenditure on housing, food, water, transport, electricity, clothing, communications, education, fuel, entertainment, travel, healthcare and other expenditure were recorded for the previous 12-month period. Per capita household expenditure adjusted by adult equivalence (AE) was used as the measure of living standard in our study [13]. Household expenditure was obtained from the household heads or the most suitable household member. Healthcare expenditure was obtained from interviewees’ medical records.

China’s healthcare system is financed through general taxation, social health insurance schemes, commercial health insurance schemes, and out-of-pocket (OOP) payments. Healthcare payments were computed using two sources: the household survey, and tax information and copayments for social health insurance, which were collected from the local statistical yearbook. General taxation is an important financing source for healthcare in China, and comes from a range of sources including excise on eating, drinking and accommodation; cigarettes, alcohol, gas, electricity, and entertainment; and other consumption taxes. Tariffs for tax were collected from the China Price Statistical Yearbook [14], and general taxation was approximated by applying tariffs for tax to the corresponding data on expenditure collected in the survey. There are no taxes specifically earmarked for health in China, so we assumed that the proportion of general taxation going to the health sector was calculated on a pro-rata basis. In 2002, tax-funded expenditure was 79.97% of government expenditure. Government expenditure on health was 4.12% of general government expenditure [15]. The proportion of household tax payments going into the health sector was therefore assumed to be 3.29%. In 2007, the proportion of household tax payments going into the health sector was estimated at 4.75% [16]. The UWBMI financing contribution was measured by applying the contribution rates to earnings of insured workers. The contribution rates for UWBMI were collected from the Gansu Statistical Yearbook [12]. Flat rate contributions were directly recorded during household interviews for those covered by CMS and NCMS. Private health insurance payments are directly obtained from the household interview. The inquiry into OOP payments involved information about health care expenditures on prescription and outpatient care paid by individuals during the latest 2 weeks before the household interview, and inpatient care paid by individuals during the previous 12 months.

### Data analysis

Unit of the finance of health care is household, with expenditures and healthcare payments aggregated to the household level. The value of household expenditure is used as the measurement of ability to pay. Household expenditure is adjusted for household size and composition to obtain an adult equivalent estimate. The number of adult equivalent household members is defined as

$${\mathrm{AE }= (\mathrm{A }+ 0.5\mathrm{K})}^{0.75}$$

where A is the number of adults (> 14 years) in the household and K the number of children (0–14 years) [17].

Contributions toward the finance of health care may redistribute disposable income of households. The types of redistribution include vertical redistribution and horizontal redistribution. The former occurs when healthcare payments are disproportionately related to ability to pay. The latter occurs when persons with equal ability to pay contribute unequally to healthcare payments. Together with reranking, vertical and horizontal redistribution are generally defined as the redistributive effect (RE) [17]. Vertical equity implies that people having greater ability to pay ought to pay more and horizontal equity implies that people with equal ability to pay should pay the same. Reranking occurs when people change rank order before and after healthcare payments.

In 1994, Aronson, Johnson and Lambert provided a decomposition method to measure the RE of income tax [3]. Later, Wagstaff and van Doorslaer applied this Aronson–Johnson–Lambert (AJL) decomposition method to decompose the change in income inequality caused by healthcare financing into a vertical, horizontal and reranking effect [4]. The extent of vertical equity, horizontal inequity and reranking calculations are usually expressed as percentages of the total RE.

The RE of healthcare finance can be calculated as the difference in the Gini coefficient caused by the healthcare payment:

$$RE\equiv G^X-G^{X-P}$$

where ${G}^{X}$ and ${G}^{X-P}$are the pre-payment and post-payment Gini coefficients, $X$denotes pre-payment income, or more generally some measure of ability to pay [18], and $P$ denotes the healthcare payment. AJL decomposition method has shown that this difference can be expressed as:

$$RE=V-H-R$$

The first term, V, measures the inequality reduction that would have been obtained if there had been no differential tax treatment. The second term, H, measures the extent of classical horizontal inequity. The third term, R, measures the extent of reranking in the move from the pre-payment distribution to the post-payment distribution, by comparing the post-payment Gini coefficient with the post-payment concentration index. If there is no reranking, R is zero.

Horizontal inequity H is measured by the weighted sum of the group (j) specific post-payment Gini coefficients, ${G}_{j}^{X-P}$, where weights are given by the product of the group’s population share and its post-payment income share, ${a}_{j}$.

$$H=\sum\limits_ja_jG_j^{X-P}$$

R captures the extent of reranking of households that occurs in the move from pre-payment to post-payment income distributions. It is measured as the difference between the post-payment Gini coefficient ${G}^{X-P}$ (which ranks households by post-payment income) and the post-payment concentration index ${C}^{X-P}$ (which ranks households by their pre-payment income):

$$R={G}^{X-P}-{C}^{X-P}$$

## Results

Tables 4, 5, 6 and 7 present the results of horizontal inequity and reranking of healthcare financing sources, and the distribution of healthcare financing sources across equivalent income deciles.

**Table 4:** Horizontal inequity and reranking of the Chinese urban health care financing system in 2002

**Decile**	**Equivalent household expenditure**	**General taxes**	**Social insurance contributions**	**Private insurance premiums**	**OOP**	**Total**
1—poorest	2.15%	2.18%	0.51%	0.03%	1.87%	1.60%
2	3.36%	3.40%	1.43%	1.35%	3.05%	2.77%
3	4.49%	4.56%	1.52%	1.42%	3.91%	3.50%
4	5.29%	5.34%	3.18%	1.95%	4.28%	4.01%
5	6.29%	6.34%	4.92%	6.62%	5.07%	5.20%
6	8.44%	8.43%	9.50%	8.51%	8.64%	8.71%
7	9.87%	9.88%	10.91%	14.32%	9.07%	9.67%
8	11.87%	11.76%	19.96%	12.74%	11.55%	12.49%
9	15.42%	15.36%	18.50%	21.05%	16.65%	17.14%
10—richest	32.81%	32.76%	29.57%	31.99%	35.91%	34.91%
total	100.00%	100.00%	100.00%	100.00%	100.00%	100.00%
g		0.003158	0.015288	0.011513	0.121500	0.151458
V		0.000011	0.001176	0.001101	0.007360	0.010280
H		0.000019	0.000246	0.000509	0.003933	0.004531
R		0.000000	0.000029	0.000538	0.005632	0.006204
RE		—0.000008	0.000901	0.000054	—0.002204	—0.000454
RE / RE		100.00%	100.00%	100.00%	100.00%	100.00%
V / RE		—148.01%	130.57%	2025.36%	—333.95%	—2264.02%
H / RE		—247.99%	27.36%	936.09%	—178.43%	—997.83%
R / RE		0.00%	3.22%	989.25%	—255.51%	—1366.18%

*OOP* out-of-pocket payment

*g* Payments as fraction of income

*V* Vertical effect

*H* Horizontal inequity

*R* Reranking

*RE* Redistributive effect

**Table 5:** Horizontal inequity and reranking of Chinese rural health care financing system in 2002

**Decile**	**Equivalent household expenditure**	**General taxes**	**Social insurance contributions**	**Private insurance premiums**	**OOP**	**Total**
1—poorest	1.95%	1.95%	1.24%	7.11%	2.23%	2.39%
2	3.64%	3.89%	9.16%	1.23%	3.28%	3.25%
3	4.73%	5.01%	4.20%	0.39%	4.54%	4.40%
4	5.51%	5.94%	3.42%	2.04%	4.53%	4.46%
5	6.68%	7.16%	1.56%	1.01%	5.32%	5.17%
6	7.61%	8.20%	9.13%	4.73%	5.85%	5.88%
7	8.41%	8.62%	10.52%	11.97%	9.63%	9.70%
8	9.98%	10.46%	13.46%	9.65%	9.04%	9.11%
9	12.43%	12.72%	9.07%	23.69%	10.98%	11.46%
10—richest	39.05%	36.05%	38.24%	38.18%	44.59%	44.17%
total	100.00%	100.00%	100.00%	100.00%	100.00%	100.00%
g		0.003045	0.000989	0.005995	0.156473	0.166504
V		—0.000309	—0.000169	0.000023	—0.002670	—0.003247
H		—0.000222	—0.000089	0.000114	0.002336	0.002201
R		0.000000	0.000000	0.000321	0.003959	0.004429
RE		—0.000087	—0.000080	—0.000412	—0.008965	—0.009877
RE / RE		100.00%	100.00%	100.00%	100.00%	100.00%
V / RE		355.21%	210.90%	—5.58%	29.78%	32.87%
H / RE		255.21%	110.90%	—27.62%	—26.06%	—22.28%
R / RE		0.00%	0.00%	—77.96%	—44.16%	—44.85%

*OOP* out-of-pocket payment

*g* Payments as fraction of income

*V* Vertical effect

*H* Horizontal inequity

*R* Reranking

*RE* Redistributive effect

**Table 6:** Horizontal inequity and reranking of Chinese urban health care financing system in 2007

**Decile**	**Equivalent household expenditure**	**General taxes**	**Social insurance contributions**	**Private insurance premiums**	**OOP**	**Total**
1—poorest	3.01%	3.37%	1.33%	3.19%	2.35%	2.14%
2	4.41%	4.74%	2.52%	2.49%	3.55%	3.25%
3	5.49%	5.63%	4.49%	3.72%	5.28%	5.01%
4	6.65%	6.77%	5.85%	3.46%	6.22%	6.01%
5	7.81%	7.81%	8.05%	8.72%	7.66%	7.81%
6	9.17%	9.33%	9.44%	13.85%	8.58%	9.08%
7	10.87%	10.83%	10.86%	14.08%	11.28%	11.29%
8	12.82%	12.92%	14.67%	19.71%	11.77%	12.94%
9	15.39%	15.34%	17.60%	11.21%	15.02%	15.53%
10—richest	24.38%	23.26%	25.19%	19.57%	28.29%	26.94%
total	100.00%	100.00%	100.00%	100.00%	100.00%	100.00%
g		0.004308	0.053441	0.009519	0.133745	0.201012
V		—0.000057	0.004064	0.000137	0.008465	0.014091
H		0.000020	0.000795	0.000299	0.002805	0.003848
R		0.000000	0.000320	0.000703	0.009125	0.012168
RE		—0.000077	0.002949	—0.000865	—0.003464	—0.001925
RE / RE		100.00%	100.00%	100.00%	100.00%	100.00%
V / RE		74.16%	137.80%	—15.82%	—244.38%	—731.83%
H / RE		—25.83%	26.94%	—34.56%	—80.96%	—199.87%
R / RE		0.00%	10.85%	—81.26%	—263.42%	—631.96%

*OOP* out-of-pocket payment

*g* Payments as fraction of income

*V* Vertical effect

*H* Horizontal inequity

*R* Reranking

*RE* Redistributive effect

**Table 7:** Horizontal inequity and reranking of Chinese rural health care financing system in 2007

**Decile**	**Equivalent household expenditure**	**General taxes**	**Social insurance contributions**	**Private insurance premiums**	**OOP**	**Total**
1—poorest	2.90%	3.03%	6.07%	0.28%	2.72%	2.67%
2	4.51%	4.65%	7.30%	0.80%	4.04%	3.94%
3	5.61%	5.69%	7.45%	0.37%	5.46%	5.15%
4	6.55%	6.85%	7.43%	4.31%	5.41%	5.45%
5	7.58%	7.70%	8.57%	5.72%	7.55%	7.45%
6	8.71%	8.62%	8.82%	4.30%	9.76%	9.27%
7	10.23%	10.07%	10.18%	11.91%	11.67%	11.59%
8	12.27%	12.26%	12.45%	13.23%	12.90%	12.89%
9	15.15%	15.26%	14.00%	15.92%	15.14%	15.16%
10—richest	26.48%	25.87%	17.71%	43.16%	25.34%	26.44%
total	100.00%	100.00%	100.00%	100.00%	100.00%	100.00%
g		0.004435	0.006671	0.012995	0.142700	0.166801
V		—0.000016	—0.000938	0.003535	0.002745	0.005874
H		0.000028	0.000083	0.000696	0.004890	0.005757
R		0.000000	0.000044	0.001286	0.009668	0.011914
RE		—0.000044	—0.001065	0.001553	—0.011813	—0.011796
RE / RE		100.00%	100.00%	100.00%	100.00%	100.00%
V / RE		36.64%	88.09%	227.61%	—23.24%	—49.80%
H / RE		—63.36%	—7.80%	44.79%	—41.40%	—48.80%
R / RE		0.00%	—4.11%	82.82%	—81.84%	—101.00%

*OOP* out-of-pocket payment

*g* Payments as fraction of income

*V* Vertical effect

*H* Horizontal inequity

*R* Reranking

*RE* Redistributive effect

### Urban areas in 2002

Table 4 shows that 15.15% of household expenditure was payments for healthcare. RE was negative (− 0.000454) for the overall healthcare financing system, suggesting that the redistribution favored wealthier households (pro-rich). It would have been 2364.02% less redistributive without differential treatment, 997.83 percentage points being the result of horizontal inequity and 1366.18 the result of reranking. General tax showed a slightly pro-rich structure because the RE value was negative (− 0.000008). It would have been 248.01% less redistributive without differential treatment, which depended wholly on horizontal inequity. RE for social health insurance was positive (0.000901) and showed a pro-poor redistribution. It would have been 30.57% more redistributive without differential treatment, the majority (27.36 percentage points) being the result of horizontal inequity. Commercial health insurance had a pro-poor effect because its RE value was positive (0.000054). The redistribution would have been 1925.36% more without differential treatment, 936.09 percentage points as a result of horizontal inequity and 989.25 percentage points from reranking. RE for OOP payments (− 0.002204) was negative, suggesting its redistribution was pro-rich. It would have been 433.95% less redistributive without differential treatment, with 178.43 percentage points from the result of reranking and 255.51 percentage points from horizontal inequity.

### Rural areas in 2002

As shown in Table 5, healthcare payments made up 16.65% of household expenditure. The healthcare financing system showed a pro-rich redistribution with a negative value for RE (− 0.009877). Horizontal inequity accounted for 22.28 percentage points, and reranking for 44.85 percentage points. This means that the healthcare financing system does not treat households with equal household expenditure equally, and households are also reranked after healthcare payments. The system would have been 67.13% less redistributive without differential treatment. General tax showed a slightly regressive structure because its value of RE was negative (− 0.000087). It would have been 255.21% less redistributive without differential treatment, which was solely from horizontal inequity. Social health insurance had a negative RE (− 0.000080), implying that it was pro-rich. It would have been 110.90% less redistributive without differential treatment, which was again solely from horizontal inequity. Commercial insurance also had a pro-rich effect with a negative RE (− 0.000412). It would have been 105.58% less redistributive without differential treatment, with 27.62 percentage points from horizontal inequity and 77.96 percentage points from reranking. RE for OOP payments (− 0.008965) was negative, suggesting its redistribution was pro-rich. It would have been 70.22% less redistributive without differential treatment, with 44.16 percentage points from horizontal inequity and 26.06 percentage points from reranking.

### Urban areas in 2007

Table 6 shows that healthcare payments made up 20.10% of urban household expenditure in 2007. The value of RE for total healthcare payments was negative (− 0.001925), showing a pro-rich redistribution. It would have been 831.83% less without differential treatment, with 199.87 percentage points to the result of horizontal inequity and 631.96 percentage points from reranking. General tax was slightly pro-rich redistributive. It would have been 25.83% less redistributive without differential treatment, which was solely the result of horizontal inequity. Social health insurance had a pro-poor redistribution, and would have been 37.80 more without differential treatment, with 26.94 percentage points from horizontal inequity and 10.85 percentage points from reranking. Commercial health insurance, however, was a pro-rich redistribution and would have been 115.82 percentage points less without differential treatment. Horizontal inequity accounted for 34.56 percentage points of the RE, and reranking for 81.26 percentage points. A much higher degree of differential treatment occurred in OOP payments, which would have been 344.38% less redistributive without differential treatment, with 80.96 percentage points from horizontal inequity and 263.42 percentage points from reranking.

### Rural areas in 2007

Table 7 shows that 16.68% of rural household expenditure in 2007 went on healthcare payments. The value of RE for total healthcare payments was negative (− 0.011796), showing that the healthcare financing system was again a pro-rich redistribution. It would have been 149.80% less redistributive without differential treatment, with 48.80 percentage points from horizontal inequity and 101.00 percentage points from reranking. General tax had a slightly pro-rich redistribution. It would have been 63.36% less without differential treatment, which was solely the result of horizontal inequity. Social health insurance was a pro-rich redistribution, and would have been 11.91% less without differential treatment, with 7.80 percentage points from horizontal inequity and 4.11 from reranking. Commercial health insurance had a pro-poor redistribution that would have 127.61 percentage points more without differential treatment. In total, 44.79 percentage points were from horizontal inequity and 82.82 from reranking. OOP payments had a pro-rich redistribution that would have been 123.24% less without differential treatment. In total, 41.40 percentage points were from horizontal inequity and 81.84 from reranking.

## Discussion

Horizontal inequity of general taxation decreased in both urban and rural areas from 2002 to 2007, indicating that it has gradually become a comprehensive tool of income redistribution in the general population. In the 1990s, the tax-levying system was not well-developed and tax avoidance was quite common in China [19]. This resulted in different financing burdens among individuals with the same income. The extent of horizontal inequity in general taxation was therefore comparatively high in 2002. In the 2000s, with improved information technology, the Chinese government enhanced tax supervision by introducing a number of tax avoidance countermeasures, such as identity certification, tax withholding and remitting, and non-cash transactions on block trading [20]. Consequently, the financing burden was equalized among people with the same income levels. The extent of horizontal inequity of general taxation was therefore significantly reduced by 2007.

Horizontal inequity was largely unchanged for social health insurance in urban areas over the period 2002–2007. Over this period, the social health insurance provider was UWBMI in urban areas. UWBMI is managed and operated by local governments, which organize universal health insurance for urban workers. The premium is jointly funded by employers and employees, and the funding amount depends on the individual’s age. Generally, employers contribute 6–8% of employees’ salaries for workers under 45 years old and 8–10% for those aged 45 or over. The employees themselves contribute 2% of their salaries [21]. Insured employees therefore pay a fixed proportion of their salary as premium, but this proportion varies slightly by age and region. More types of workers were gradually covered by UWBMI over the period, including migrant workers and those in private enterprises, social organizations, and foreign-invested enterprises, but the premium-setting policy was the same for everyone. This resulted in similar financing contributions from people with the same income levels. Horizontal inequity of UWBMI came from the different ages and regions involved, and was therefore both stable and at an acceptable level.

Horizontal inequity dramatically decreased for social health insurance in rural areas during the period 2002–2007. In 2002, the social health insurance provider was the remaining CMS, and this had been substituted by NCMS since 2003. Both had flat rate contribution schemes, so the financing contribution was the same in absolute terms for all insured individuals. The extent of horizontal inequity of CMS was quite high in 2002 because coverage was less than 10% (Table 1) and the horizontal inequity stemmed from the discrepancy between the number of covered and the uncovered. By 2007, NCMS coverage was nearly 90% and the horizontal inequity therefore significantly decreased during the period. Commercial health insurance did not play an important role in healthcare financing system because China’s authorities decided to achieve universal health coverage through social health insurance [22, 23]. Commercial insurance therefore only accounted for approximately 3% of the total healthcare payments made in the last decade [24]. Insured individuals purchased different types of insurance from different providers and the horizontal inequity was comparatively high.

During the period 2002–2007, horizontal inequity in OOP payments dramatically decreased in urban areas, but slightly increased in rural areas. OOP payments are post-paid, and the change in their horizontal equity may be explained by pre-paid payments, such as general taxation, and social and commercial health insurance. In urban areas, the horizontal inequity in OOP payments was reduced by tax avoidance countermeasures, the UWMBI’s premium-setting policy and the decreasing horizontal inequity in commercial health insurance. In rural areas, general taxation and social health insurance both decreased, but horizontal inequity in OOP payments was affected by the increase in horizontal inequity of commercial health insurance, which resulted in a slight increase in horizontal inequity of OOP payments. In both urban and rural areas, the extent of reranking of OOP payments increased over the period. This suggested that the rank order of individuals who paid for medical care through OOP payments decreased significantly, and some even dropped below the poverty line.

Policymakers took the expansion of health insurance schemes seriously in China. UWBMI, and NCMS, were either established or extended during this period. This was expected to decrease the heavy dependence on OOP payments and reduce their adverse impact on household income. The expansion of health insurance schemes was designed not only to improve access to basic medical care, but also to provide adequate and effective financial protection. However, social health insurance focused on ensuring wide universal coverage, not depth of risk pooling.

Health insurance was administered and implemented at county level. The county government’s top priority was fund security, and a deficit in fund pooling was not encouraged. This resulted in a very strict compensation policy for insured patients. For example, only services from contracted hospitals and pharmacies were eligible for reimbursement. Reimbursement depended on the provincial health insurance list, but many types of medicines and medical services, especially the more expensive items, were not covered. Patients had to pay for these medicines and medical care. Even the costs of the medical services in the list were not fully reimbursed. It was found that the level of NCMS deductibles was low, but so were the co-payment rate and ceiling [25]. The costs of catastrophic illness requiring hospitalization were often not reimbursed because of underfunding [26, 27]. As a result, many urban and rural residents still faced high economic risk of diseases and dramatic changes in household economic rank were unavoidable following high healthcare payments. OOP payments as a fraction of income (g) were far larger than all other healthcare payments. This indicated that the impact of horizontal inequity in OOP payments was much larger than in other healthcare payments. The horizontal inequity of the total healthcare payments was largely dominated by OOP payments.

Between 2002 and 2007, therefore, horizontal inequity and reranking decreased in urban areas and increased in rural areas. In cities, horizontal inequity of social health insurance stayed broadly the same, but there was a significant decrease in horizontal inequity of commercial health insurance. This indicated that more and more urban residents chose social health insurance. The tax-levying policy also decreased the horizontal inequity in general taxation. These actions resulted in the decreasing level of horizontal inequity of total health finance in urban areas. In rural counties, the horizontal inequity of general taxation and social health insurance also decreased significantly, but OOP payments were the driving force behind an overall increase in horizontal inequity of total health finance. This result indicates that, although NCMS coverage reached nearly 90% over the period between 2002 and 2007, horizontal inequity of total healthcare finance was not reduced. This suggests that population coverage was just one dimension in the expansion of health insurance coverage. Updating of cost coverage and service coverage are also key elements in reform of health insurance schemes. Designing a rational financing mechanism for individuals within income groups and between income groups can, however, help to reduce horizontal inequity.

Some limitations of our study must be acknowledged. A limitation was that the data were collected from a single province in China, and the results obtained did not entirely represent the characteristics of national healthcare financing. Another limitation was that, as with other cross-sectional studies, we cannot conclude that the observed changes in horizontal inequity of healthcare financing had been caused by health insurance reform. Other uncontrollable factors would affect financing equity, such as regional economic development, health literacy, and quality of health technology.

## Conclusion

Social health insurance schemes may be best funded through pro rata contributions, rather than flat rate contributions. General taxation and social insurance reduced the horizontal inequity of healthcare finance in China, but it was still strongly affected by OOP payments. Increasing the benefits package of social health insurance would be helpful to reduce the horizontal inequity of healthcare finance still further.

## Data Availability

The datasets used in the current study are not publicly available due to the confidential policy but are available from the corresponding author on reasonable request.

## Abbreviations

AE: Adult equivalence
AJL: Aronson–Johnson–Lambert
CMS: Cooperative Medical Scheme
NCMS: New Cooperative Medical Scheme
NHSS: National Health Services Survey
OOP: Out-of-pocket payment
RE: Redistributive effect
UWBMI: Urban Workers Basic Medical Insurance

## References

[1] China’s Ministry of Health (2004). An analysis of the third national health services survey.

[2] China’s Ministry of Health (2009). An analysis of the fourth national health services survey.

[3] Aronson JR, Johnson P, Lambert PJ (1994). Redistributive effect and unequal income tax treatment. Econ J.

[4] Wagstaff A, van Doorslaer E (1997). Progressivity, horizontal equity and reranking in health care finance: a decomposition analysis for The Netherlands. J Health Econ.

[5] Chen M, Chen W, Zhao Y (2012). New evidence on financing equity in China's health care reform–a case study on Gansu province, China. BMC Health Serv Res.

[6] Elwell-Sutton TM, Jiang CQ, Zhang WS, Cheng KK, Lam TH, Leung GM, Schooling CM (2013). Inequality and inequity in access to health care and treatment for chronic conditions in China: the Guangzhou Biobank Cohort Study. Health Policy Plan.

[7] Chen M, Zhao Y, Si L (2014). Who pays for health care in China? The case of Heilongjiang province. PLoS One.

[8] Yang W (2013). China's new cooperative medical scheme and equity in access to health care: evidence from a longitudinal household survey. Int J Equity Health.

[9] Gerdtham UG, Sundberg G (1998). Redistributive effects of Swedish health care finance. Int J Health Plann Manage.

[10] Bilger M (2008). Progressivity, horizontal inequality and reranking caused by health system financing: a decomposition analysis for Switzerland. J Health Econ.

[11] Cavagnero E, Bilger M (2010). Equity during an economic crisis: financing of the Argentine health system. J Health Econ.

[12] Gansu Provincial Bureau of Statistics (2010). Gansu Statistical Yearbook 2010.

[13] Wilde PE (2000). The analysis of household surveys: A microeconometric approach to development policy. Am J Agr Econ.

[14] National Bureau of Statistics of China (2008). China Price Statistical Yearbook 2008.

[15] National Health Development Research Center (2003). China National Health Accounts Report 2003.

[16] National Health Development Research Center (2008). China National Health Accounts Report 2008.

[17] O'Donnell O, Doorslaer Ev, Wagstaff A, Lindelow M (2007). Analyzing Health Equity Using Household Survey Data: A Guide to Techniques and their Implementation.

[18] Wagstaff A, van Doorslaer E (2003). Catastrophe and impoverishment in paying for health care: with applications to Vietnam 1993–1998. Health Econ.

[19] Kim H-K (2004). The Politics of Fiscal Standardization in China: Fiscal Contract vs. Tax Assignment. Asian Perspective.

[20] Sun Z, Chang CP, Hao Y (2017). Fiscal decentralization and China's provincial economic growth: a panel data analysis for China's tax sharing system (vol 51, pg 2267, 2017). Qual Quant.

[21] Huang F, Gan L (2017). The Impacts of China's Urban Employee Basic Medical Insurance on Healthcare Expenditures and Health Outcomes. Health Econ.

[22] Wang HQ, Liu ZH, Zhang YZ, Luo ZJ (2012). Integration of current identity-based district-varied health insurance schemes in China: implications and challenges. Front Med.

[23] Li Y, Wu Q, Xu L, Legge D, Hao Y, Gao L, Ning N, Wan G (2012). Factors affecting catastrophic health expenditure and impoverishment from medical expenses in China: policy implications of universal health insurance. Bull World Health Organ.

[24] National Health Development Research Center (2011). China National Health Accounts Report 2011.

[25] Brown PH, Theoharides C (2009). Health-seeking behavior and hospital choice in China's New Cooperative Medical System. Health Econ.

[26] Jing Z, Chu J, Imam Syeda Z, Zhang X, Xu Q, Sun L, Zhou C. Catastrophic Health Expenditure among Type 2 Diabetes Mellitus Patients: a Province-wide Study in Shandong, China. J Diabetes Investig. 2019;10:283–9. 10.1111/jdi.12901 PMC6400173 30044060

[27] Sylvia S, Xue H, Zhou C, Shi Y, Yi H, Zhou H, Rozelle S, Pai M, Das J (2017). Tuberculosis detection and the challenges of integrated care in rural China: a cross-sectional standardized patient study. PLoS Med.

